# Craniofacial Anomaly Association with the Internal Malformations in the Pediatric Age Group in Al-Fallujah City-Iraq

**DOI:** 10.1155/2020/4725141

**Published:** 2020-08-18

**Authors:** Thaer M. Farhan, Basim A. Al-Abdely, Abdulrahman N. Abdullateef, Abdulhameed S. Jubair

**Affiliations:** ^1^Department of Human Anatomy, College of Medicine-Al-Nahrain University, Baghdad-, Iraq; ^2^Pediatrics Department, Fallujah Teaching Hospital for Maternity and Childhood, Fallujah-, Iraq; ^3^College of Medicine, University of Fallujah, Iraq

## Abstract

**Objectives:**

To estimate the prevalence of craniofacial anomalies among Iraqi people and its association with other congenital malformations.

**Methods:**

A hospital-based cohort study. It was conducted in Iraq, Fallujah city from Jan 2019-April 2019. The pediatric age group below 16 years attending the consultation clinic.

**Results:**

The prevalence rate of craniofacial anomalies was 2%. There were 43 (54%) males and 37 (46%) females. A 55 cases (69%) out of total 80 cases have an association with other internal congenital malformations, and 25 cases (31%) have no association. Those associated internal malformations were categorized according to their types into congenital heart disease 33(60%), Renal diseases 9 (16%), CNS anomalies 8(15%), and GIT anomalies 5(9%).

**Conclusions:**

Craniofacial anomalies showed a relatively higher prevalence rate in comparison to other studies worldwide. It was found that the majority of craniofacial anomalies might be associated with other congenital systemic malformations. Furthermore, the necessary actions to identify the frequency and risk factors associated with craniofacial anomalies in the Iraqi population are emphasized to put a better strategy to establish future preventive programs and treatment.

## 1. Introduction

Craniofacial anomalies (CFA) are important pediatrics health problems. They are a major cause of infant mortality and childhood morbidity [1]. CFA primarily affected the cranium and facial bones and associated with the development of the pharyngeal arches [2]. These abnormalities are varied from mild to severe that might be life-threatening and need immediate surgical intervention [3].

Craniofacial syndromes can be divided into groups of categories like premature fusion of the cranial sutures (craniosynostosis) and those related to clefts malformation. The most common craniofacial anomalies in pediatrics: Crouzon, suture synostosis, microsomy, cephalic abnormalities, and midfacial clefts [4, 5].

These developmental disorders can lead to various health consequences that affect the airways, facial appearance, cerebral development, hearing, sight, dentition, speech, and psychological well-being of these patients [5–7].

CFA might be associated with diverse genetic and environmental factors. There is a significant psychological impact of altered facial and dental appearance in patients with craniofacial anomalies compared to controls [8–10].

CFA can be caused by a combination of genes as a child may receive a particular combination of gene(s) from one or both parents, or there may be gene alteration during pregnancy time that may cause craniofacial defects. Environmental cause is not fully understood. However, environmental exposures may play a role, especially in combination with genetic abnormalities [1]. Studies have shown that females with folic acid deficiency, or taking a folate-deficient meal, may be at risk of developing congenital birth defects, including cleft lip and/or cleft palate [3]. Microdeletions of gene may be associated with heart defects and craniofacial deformity in humans [11].

The main types of the CFA:

1. Cleft lip and/or cleft palate. The common congenital craniofacial defects seen at birth [1, 3]
2. Craniosynostosis. one or more cranial sutures prematurely fused. It is associated with diverse environmental and genetic factors. Whereas isolated single-suture synostosis is usually sporadic and nonfamilial. [8]
3. Hemifacial microsomia. The tissues in unilateral face are underdeveloped, affecting primarily the ear, mouth, and mandible areas. Sometimes, the face can be affected bilaterally and the skull may be involved, as well as the face [1, 3]
4. Vascular malformation. A birthmark or growth, present at birth that is composed of blood vessels and can cause functional or aesthetic problems [1]
5. Hemangioma is an abnormally growing blood vessel in the skin that may be present at birth (faint red mark) or appear in the first months after birth [3]
6. Deformational (or positional) plagiocephaly. Deformed shape of the cranium from repeated pressure to a fixed point of the skull. Plagiocephaly literally means “oblique head” (Greek terminology) “cephale” for head. [1, 3, 11]
7. External ear deformities are classified using the system described by Meurman and modified by Marx [12, 13]

Grade I: Mild hypoplasia, with obvious malformation, but with all structures present.

Grade II: Atresia of the external auditory canal.

Grade III: Absent auricle, the lobular remnant is anteriorly and inferiorly displaced [14].

(8) Dysmorphic type of skull shows a spherical big head with a medium-width nostrils, rounded orbital edges, prominent cheekbones, shallow canine fossae, moderate prognathism, absent brow ridges, giant skull sutures, prominent zygomatic bones, wide and flat nasal bridge, less prominent nasal spine, shovel-shaped upper incisor teeth (scooped out behind), moderately wide palate shape, arched sagittal contour, wide, and flat face [15, 16]

The congenital defects affect 2-3% of all children and about 1% of them have syndromes or multiple deformities [1]. Syndromes consist of multiple malformations that could be etiologically or pathogenetically related or both. Approximately 30% of of the total cases that include cleft lip and/or cleft palate are syndromic, which need more research to determine the etiology of them [1].

Research suggests that associated anomalies occur with a frequency of 44% to 64% in patients with orofacial clefts [17]. CFA, except cleft lip and palate, occurs in 1 in 1600 live births in the United States of America (USA) including mandible defects, malformed teeth and deformity in craniofacial bone ossification. Clefts deformitiesoccur in Asian people more than African people [1].

According to the WHO report in 2002, many factors contribute to cleft conditions, among them being heredity, prenatal nutrition, drug exposure, and other environmental factors. Little et al. 2014 stated that Maternal smoking during pregnancy has been linked consistency with increased risk of both cleft lip with or without cleft palate and isolated cleft palate, with a population–attributable risk as high as 20%, while Botto et al. 2002 stated that Maternal use of multivitamin supplements in early pregnancy has been linked to decrease risk of OFC (orofacial cleft); in a meta-analysis [1, 18, 19].

Cohen 1978 explained other associated anomalies occur with a frequency of 44% to 64% in patients with clefts, while Zhang 2001 described, interestingly; Interestingly, a similarly high prevalence rate for CL/P seems to exist in the ethnic population of Tibet at an almost equally high altitude [1, 17].

Kohli SS. and Kohli VS. 2012 explained that several genes causing syndromic CLP have been discovered. Three of them participated in causing X-linked cleft palate, cleft lip/palate–ectodermal dysplasia syndrome, while Pegelo et al. 2008 stated that Mutations of the IRF6 gene are not a common cause for cleft predisposition in Swedish NSCLP (nonsyndromic cleft palate) families [20, 21].

Rosano and Mastroiacovo 2001; Mossey and Little 2002 explained that cleft lip with or without cleft palate is the highest prevalence rate (2.28 per 10 000) reported in the world is that of Bolivia; Mossey and Little 2002 mentioned there are high prevalence rates of CL/P in Europe are reported from northern than from southern countries, while Kondo 1987 reported that there is a low prevalence of CL/P in Japan and of CP in China reported by Xiao 1989 [1].

According to Oliveira 2008, the most frequently observed craniofacial deformities in pediatrics are small nose, low nasal bridge, abnormal shape palate, splitted uvula, maldeveloped mandible, cleft lip, incomplete lip closure, hypotonic lips, fissured tongue, slow tongue movement, and changes in temporary and permanent dentation [22].

Castilla, Lopez-Camelo, and Campana 1999 explained the role of both environmental (chronic hypobaric hypoxia from altitude) and genetic etiologic factors, and their interactions are still unknown. Alharbi et al. (2017) explained that, a detailed knowledge of the genetic processes is involved in the development of craniofacial structures and so, The development of novel clinical therapies for craniofacial abnormalities, such as clefts and tooth agenesis, depends very much on genetic information [1, 23].

Edward 2014 stated that the most common syndrome associated with craniosynostosis include crouzon, Apert, Muenke, and Saethre Chotzen syndromes [24].

In 2010, a pilot study of congenital anomaly incidience rate at birth was carried out in Al-Fallujah General Hospital, Fallujah, Iraq, between November 2009 and October 2010. The study included 291 infants and children with congenital anomalies recorded at birth and constituted about 4.8% of all live births during the study period (291/6049). The study results showed the following congenital anomalies: 113 cases of congenital anomalies were in the heart and circulatory system, 72 cases in the nervous system, 40 cases in the digestive system, 9 cases in genitourinary, 6 cases in the ear, face and neck, 7 cases in respiratory, and 30 cases were down syndrome [25].

It has been stated that the fetal cranial and facial examination during prenatal ultrasound studies and a thorough search for other associated abnormality has great importance [26].

The Aim:

1. To estimate the prevalence of craniofacial anomalies among population in the area of study
2. To assess the association between the development of craniofacial anomalies with other congenital internal malformations (cardiac, respiratory, GIT, brain, and renal) in the pediatric age group, in Al-Fallujah city, Iraq

## 2. Materials and Methods

### 2.1. Design, Population, Settings

This clinical study was a prospective cohort study design. It was conducted in Al-Fallujah's maternity and childhood teaching hospital in Al-Fallujah city between Jan 2019 and April 2019. Infants and children below 16 years of age attending the consultation clinic were included in the study.

Inclusion criteria: any infant or child, male or female, from Anbar province, has any type of craniofacial deformities, congenital in origin, orsince birth, single, or multiple, mild_moderate_sever.

Exclusion criteria: any infant or child has craniofacial deformity not congenital in origin, might be traumatic or acquired during their lives.

**Figure 1:**
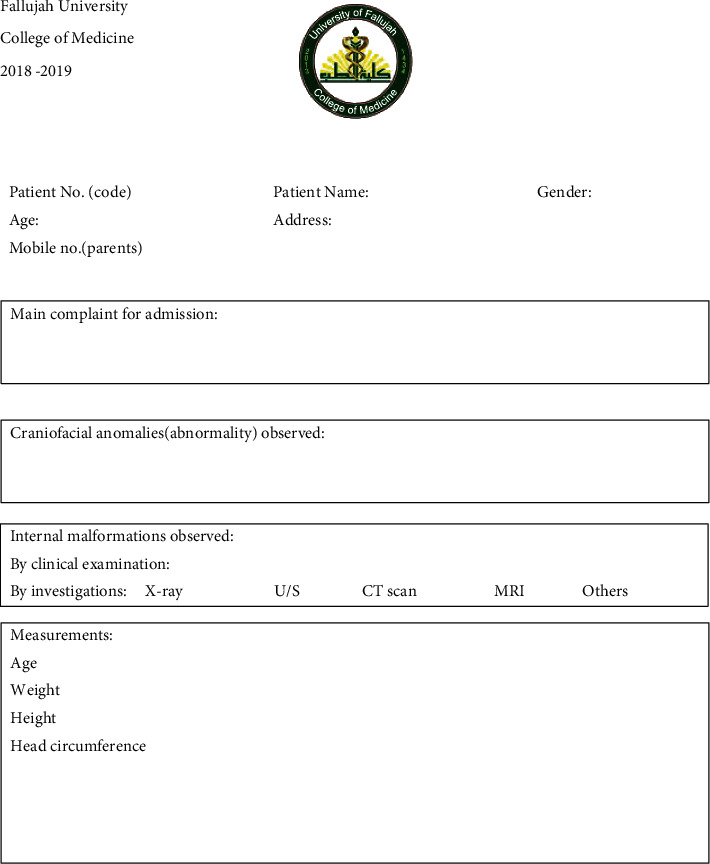
Questionnaire list of the study.

The sample was convenient, when every infant and child attended the consultation clinic at the hospital would be examined, and the data is collected randomly then including most of those patient cases with craniofacial abnormalities in the study. Using a prepared list of questionnaires that included demographic characteristics and medical history as shown in Figure 1.

Ethics: the study protocol acquainted with the ethical guidelines and requirements of the “World Medical Association” Declaration of Helsinki. An oral informed consent was obtained from each patient parents.

### 2.2. Outcome

The main outcome:

1. Prevalence of the craniofacial anomalies among pediatric patients seen in the hospital
2. Association between the development of craniofacial anomalies and other congenital malformations in the same patient and its frequency

The presence of internal malformations was confirmed by the following maneuvers:

1. Clinical examination to detect any clinical finding which gives a clue for internal cardiac, respiratory, GIT, as well the CNS congenital malformations
2. Measure the head circumference
3. X-ray of the chest to detect any related malformations
4. US (ultrasound examination) for the abdomen to detect any abnormality of the abdominal organs (like GIT, renal malformation)
5. Echocardiography to discover any associated CVS anomalies
6. CT (Computed Tomography) of the brain, if feasible

Data analysis: After collection of the data, they were analysed to measure the prevalence of the craniofacial anomalies; Excel-aided analysis and statistics are used to describe the prevalence of craniofacial anomalies and their categorization into types according to their association with other congenital malformation, their incidence, and frequency.

## 3. Results

The study was carried out on (4000) pediatric patients examined at the hospital clinics, during the period of study. Out of this number, there were (80) cases of children with craniofacial anomalies (CFA) collected at Al-Fallujah's maternity and childhood teaching hospital, in Fallujah city. These 80 CFA cases were included in the statistical analysis of the study.

**Figure 2:**
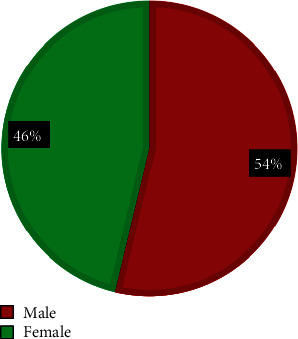
Pie chart shows a male : female ratio in the CFA (craniofacial anomalies).

**Figure 3:**
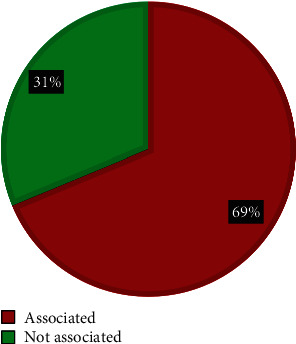
Pie chart shows the prevalence of CFA (craniofacial anomalies) and its association with internal malformations.

The prevalence of the CFA in the current study was 2 in each 100 patients examined(2%) of all pediatrics. Among these CFA cases, there were 43 (54%) males and 37 (46%) females, ranging in age from 1 day to 16 years. Of them, there were 55 cases (69%) that have an association with other internal congenital malformations and 25 cases (31%) that have no association, as shown in (Table 1 and Figures 2 and 3).

**Figure 4:**
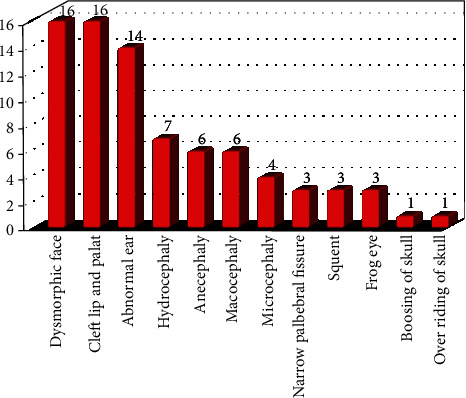
Frequency of craniofacial anomalies according to their types.

In the current research, the craniofacial malformations can be categorized in order of their frequency as follows: dysmorphic face 16 (20%), cleft lip and palate 16 (20%), followed by abnormal shape ear 14 (17.5%), then hydrocephaly 7 (8.7%), followed by anencephaly and macrocephaly 6 (7.5%) for each, then microcephaly 4 (5%), then narrow palpebral fissure 3 (3.8%), squint 3 (3.8%), frog eye 3 (3.8%), boosing of skull 1 (1.2%), overriding of skull 1 (1.2%), as shown in (Table 2 and Figure 4).

**Figure 5:**
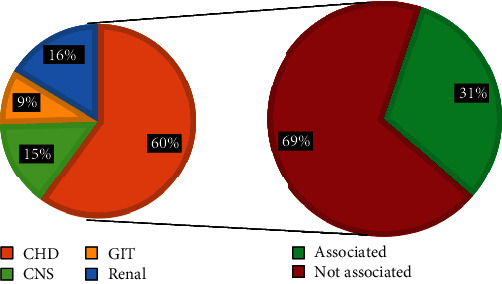
Incidence of internal malformations according to their types and frequencies.

The internal malformations which were detected during the present study showed the following frequency: congenital heart disease 33 (60%), followed by Renal diseases 9 (16%), CNS anomalies 8 (15%), then GIT anomalies 5 (9%), as shown in Figure 5.

The relatively high frequency of craniofacial anomalies, namely, dysmorphic face, cleft lip and palate, and abnormal ear shape can be further evaluated according to their correlation with internal malformations.

**Figure 6:**
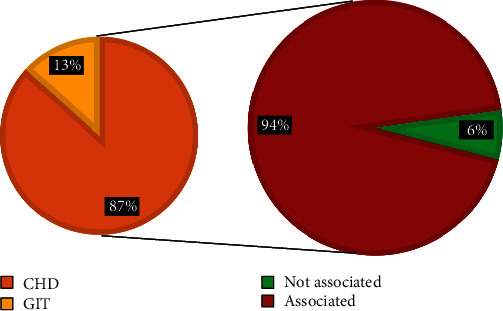
Shows dysmorphic face and its associated internal malformations.

**Figure 7:**
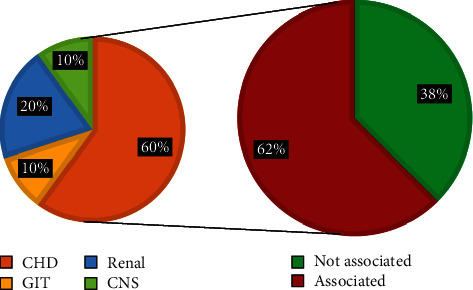
Shows the cleft lip and palate and its associated internal malformations.

**Figure 8:**
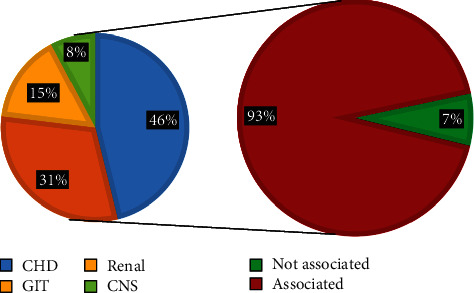
Shows the correlation of abnormal external ear shape with internal anomalies and the frequency of the internal malformations.

1. Regarding the dysmorphic face (16 cases), there were 15 cases (94%) associated with internal malformations and only 1 case (6%) not associated, of the associated cases; there were 13 cases (87%) associated with congenital heart diseases and the remaining 2 cases (13%) were associated with GIT anomalies, see Figure 6
2. For the cleft lip and palate cases (16 cases), there were 10 (62%) that were associated with internal malformation while 6 cases (38%) not associated, of the correlated; there were 6 cases (60%) associated with congenital heart disease, 2 cases (20%) with renal malformations, 1 case (10%) with GIT malformation, and 1 case (10%) with CNS malformation. See Figure 7
3. For the abnormal ear shape (14 cases), 13 cases (93%) were associated with internal malformations while only 1 case (7%) not associated, of the associated cases; there were 6 (46%) cases associated with renal malformations, 4 (31%) associated with CHD, 2 cases (15%) associated with GIT malformations, and 1 case (8%) associated CNS malformations, as shown in Figure 8

**Figure 9:**
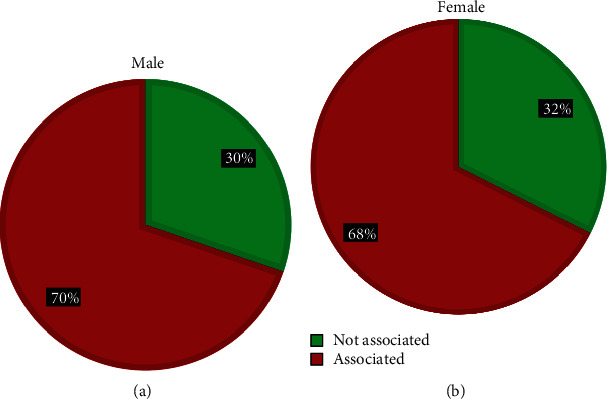
Describe cases of CFA (craniofacial anomalies) and association with internal malformations according to the types and frequencies. (a) for Male and (b) for Female.

**Figure 10:**
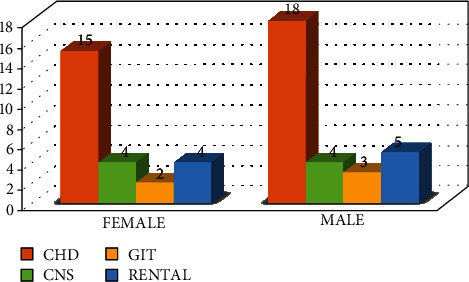
Describe male and female children cases of CFA (craniofacial anomalies) and association with internal congenital malformations.

According to the gender of the patients, in male children, there were 30 (70%) that have an association with internal malformations, and 13 (30%) have no association, while in the female children, there were 25 (68%) that have association with internal malformations, and 12 (32%) without, as seen in (Figures 9 and 10).

## 4. Discussion

Craniofacial anomalies (CFA) are congenital musculoskeletal disorders, which primarily affect the cranium and facial bones. Congenital anomalies (CA) are a major cause of infant mortality and childhood morbidity [1, 23]. They are congenital and have numerous variations. Some are mild and some are severe and need surgery. They are related to the embryonic development of the pharyngeal arches [27, 28].

The study results showed the approximate prevalence rate of the craniofacial anomalies in Iraq, particularly in the western area of Iraq, which seems to be a little bit higher rate; it was found that 2% of the pediatric population might be born or later develop CFA of different types and degrees while the congenital anomalies prevalence rate reported by WHO in 2010 was between 2-3% in all babies, whereas the prevalence rate of CFA between 0.06-0.14% [1]. This might alarm the health professionals to be more serious about the increasement in a number of such disorders in the community. In addition to that, the study described an evident association between craniofacial anomalies incidence with other internal malformations when 69% of the cases included in the study were associated with the occurrence of internal malformation in different systems, although, still need more large sample number to confirm the significance of this association and try to perform more statistical analysis. Such association might be due to many etiological, epidemiological, or environmental factors that may contribute to, for instance, the complex socioeconomic status of the general population in Iraq and Fallujah city in particular, or it might be due to the shortage of medical services especially after the military operations in Iraq since 2003, where a lot of hospitals was lacking the primary requirements for being in service which might lead to poor health among people. In addition to that, many genetic alteration and mutation is expected due to chemical weapons that been used from the second gulf war onward [25].

Environmental exposures may play a role in the development of craniofacial anomalies, especially in combination with genetic abnormalities, like folic acid deficiency [1, 2]. In the current study, most of the cases included were from families having difficult socioeconomic conditions, where their dietary supplements of the essential vitamins and minerals, important for the development of embryos, are deficient.

In the current study, the craniofacial malformations that are included in the study randomly have a variation in their frequency according to their types, for instance, the dysmorphic face, cleft lip and palate, and abnormal external ear shape have a high frequency rate consequently, while the least frequency was with skull and eye deformities, this may agree with other works that state; Cleft lip with or without cleft palate (CL/CP) is one of the most common structural birth defects [29].

Castilla et al. 1999 explained the role of both environmental and genetic (Mongolic Amerindian ethnicity) etiologic factors, and their interactions are still unknown; this might contribute to the Pathophysiology of craniofacial anomalies in Fallujah city since the frequent military battles and operations and a lot of weapons and missiles are used which may lead to pollution of the environment and water sources and contribute to the etiology of CFA [30]. Additionally, almost all the studies reviewed agreed and supported the point that congenital malformations were occurring more commonly in consanguineous couples than nonconsanguineous couples [31, 32]. From Figure 5, which describe the frequency of internal malformations detected in the study which are associated to CFA, one can see that congenital heart disease is the most frequent entity, may be due to its strong association to dysmorphic face anomaly, and this category may include down syndrome cases, which has got a known association to congenital heart diseases. The researchers here try to state that the main idea in the current study is to correlate between the incidence of CFA and its association to the occurrence of internal malformation like CHD regardless if they are syndromic or not to elaborate on the clinical implications of this association.

Craniofacial anomalies (CFAs) are a highly diverse group of complex congenital anomalies. Over many years, efforts have been made to record the frequency of birth defects [26]. Data on the frequency of CFAs are still shortened in many parts of the world, particularly in Africa, Asia, and Eastern Europe [27].

The study was conducted among pediatric age group with craniofacial anomalies with male: female ratio (1.2 : 1) which agree with other related works in the same field, it might be due to environmental exposures, genetic predisposition, which requires more research on genetic polymorphisms, and genetic environment interaction [12, 13].

There is a clear association between craniofacial anomalies and internal malformations in which the result was 69%. Most of them were dysmorphic face 16 (20%) among these were 15 (94%) correlated with internal malformations and 1 case (6%) without. According to the National Down Syndrome Society (NDSS), In the United States of America, 1 in every 700 live births is born with down syndrome. It is the most common genetic disorder in the United States [33].

Cleft lip and palate cases show association with internal malformation about in 62%, the most common associated malformation was the congenital heart disease (60%), may be due to chromosomal syndrome, then followed by renal anomalies 20%, GIT malformation 10%, and CNS malformation 10% [34]. These syndromic conditions might involve a clinically significant structural and/or numerical chromosomal abnormality. According to WHO reports, there were about 20% of infants born with orofacial clefts have associated other congenital malformations. The study of associated anomalies is useful in identifying pathogenetically homogenous patterns of malformations and participate in the determination of the etiologic studies and better public health monitoring. In the current study, the figures were high which should bring the attention of the health care providers for the increasing risk of developing CFA with or without associated malformations [1].

In neighbouring countries like Jordan, a study carried out in 2008 on cases of cleft lip and palate and its associated malformation stated that 47% of clefts cases were associated with congenital heart disease, 13% with skeletal malformation. 10% with renal malformation while in the present study, the figures were high, 60% of the clefts cases associated with congenital heart diseases, 20% with renal malformation. Another study carried out in Jordan 2008 stated that cleft lip and palate cases were associated with GIT malformation in 2.3% on the contrary, the current study stated that 10% of clefts cases are associated with GIT malformations. [35]

There are several factors that play a role in the embryonic development of the CNS and the facial structures. Thus, the etiology of CNS anomalies in CFA may be a defect in the mesodermal or neural crest cell migration [27, 28, 36, 37].

The incidence of external ear abnormality (shape, position) in the current study was 17.5% of the total number of CFA in the study, 93% of them were associated with internal malformation, the most common associated malformations were renal 46%, congenital heart disease 31%, GIT 15%, and CNS 8%, as shown in Figures 8 and 9. This will signify the role of clinician when detecting a patient with congenital ear defects and should send him for further investigations to exclude or confirm the presence of associated internal malformations. This congenital malformation may be multifactorial since it affects more than one region of the developing embryos or even due to exposure to severe teratogenic agent which lead to it, like retinoic acid (retinol). Deficiencies or resorptions of the infraorbital region and in the mandible, abnormalities of the secondary palate, and external ear dysmorphology were observed due to the teratogenic effect of retinoic acid [38].

There is an important finding in the current study that should be taken in consideration, that some of the craniofacial anomalies were associated with more than one internal malformation. For instance, the abnormal external ear shape was associated with brain atrophy and VSD in the same case; the macrocephaly was associated with meningocele and VSD; these cases may give a clue to understand the etiology which might be syndromic or genetic defects in nature. Researches on syndromic genes and their molecular pathways will provide a better understanding of human craniofacial pathology [39, 40]. There is no literature at all on such associations between CFA and other congenital malformations from Saudi Arabia [12].

There are many associations between craniofacial malformations and genetic syndromes that have been observed and studied. With this in mind, it is greatly beneficial for individuals with CFA such as orofacial clefts, OFCs; to consult a clinical geneticist. With the advancement of genetic testing, it allows for the determination and confirmation of an expanding number of genetic syndromes with known etiology. As a result, patients will be able to better understand the origin of their craniofacial malformations, other medical problems that they may be at risk for, and the recurrence risk for future pregnancies [41].

The consanguineous marriage among Iraqi families may be a contributing factor for a high incidence and prevalence of CFA and their association with other congenital internal malformations. A study completed in Saudi Arabia in 2009 showed a statistically significant association between consanguinity and occurrence of craniofacial anomalies [42, 43].

Indeed, the exact number of people with craniofacial anomalies in Iraq and the neighbouring countries like Jordan and Saudi Arabia is unknown due to lack of the registry system of birth defects and the absence of national surveys on the topic.

The study strength and importance are being the first work that described the prevalence of development of CFA in the region of the middle east, although some studies in Iraq and neighbouring countries have dealt with specific types of craniofacial anomalies but did not tackle them all, which would raise the attention of health professionals and providers to search more about this problem [12, 25].

The study has some limitations like

1. Despite the relatively large number of CFA cases in the area, there was no well-organized registry system
2. The unfeasibility of more advance screening tests like doing karyotype for those patients to detect the chromosomal disorders and genetic mutation to describe the etiological factors participated in the development of CFA
3. Some cases demanded to be examined by more advance tools like MRI, angiography, which was not so easy and not feasible most of the time

To the best of our knowledge, there are no previous studies exclusively on craniofacial anomalies, either on their incidence or on their etiology, from Iraq. Thus, this study may provide ground work for additional etiologic studies, including genetic studies in Iraq, and additionally, further work can reevaluate and study CFA in more specification and statistical analysis.

## 5. Conclusions

1. High prevalence rate of craniofacial anomalies in comparison to the other works worldwide
2. Majority of CFA might be associated with other congenital internal malformations
3. Craniofacial anomalies more common in male than female
4. Some of craniofacial anomalies detected are syndromic in nature since they were associated with more than single malformation in the same patient
5. The most common associated internal malformation was the congenital heart disease followed by renal malformations
6. All cases of craniofacial anomalies necessitate further investigation to detect any associated unnoticed malformations in other systems

## Figures and Tables

**Table 1:** Prevalence of CFA (craniofacial anomalies) in the study.

Total population	Cases with CFA	Cases without CFA	Prevalence
4000	80	3920	2%

**Table 2:** Distribution of CFA (craniofacial anomalies) cases and associated congenital malformation according to type and frequency.

CFA cases	Not associated with IM	Associated with IM	CFA associated with IM			
			Associated with CHD	Associated with renal	Associated with CNS	Associated with GIT
80	25	55	33	9	8	5
	31%	69%	60%	16%	15%	9%

IM: internal malformations; CDH: congenital heart disease; CNS: central nervous system; GIT: gastrointestinal tract.

## Data Availability

The data that support the work findings are available from the corresponding author (Thaer M. Farhan) upon reasonable request.

## Abbreviations

CFA: Craniofacial anomalies
CA: Congenital anomalies
FGRG: Fibroblast growth factor receptor
CP: Cleft palate
CL: Cleft lip
OC: Oral clef
NSCLP: Nonsyndromic cleft palate
OFC: Orofacial cleft
HC: Head circumference
MCOA: Main complain of admission
IM: Internal malformation
ASD: Atrial septal defect
VSD: Ventricular septal defect.

## Additional Points

*Declaration of Interest*. The field of congenital defects and malformations is of special consideration in our sector of health care in Iraq due to many factors that may play role in increasing the incidence rate of development of congenital anomalies, namely, the environmental, socioeconomic, health, and genetic factors. Iraq is one of the countries in the region suffering from bad and difficult socioeconomic and health conditions during the period after events of 2003 that put a burden on the community, hence, a lot of health and medical problems have been appearing more evident and need more researches and scientific work to estimate the etiology behind that.
